# Electrophysiological Signatures of Planned and Unplanned Continuous Movement Termination in Parkinson’s Disease

**DOI:** 10.1523/ENEURO.0286-25.2025

**Published:** 2025-10-28

**Authors:** Apoorva Karekal, Kelsey Schultz, Alexander P. Rockhill, Hae-Young Hawong, Sara Weston, Svjetlana Miocinovic, Nicole C. Swann

**Affiliations:** ^1^Departments of Human Physiology, Eugene, Oregon 97403; ^2^Biology, University of Oregon, Eugene, Oregon 97403; ^3^Department of Neurological Surgery, Oregon Health & Science University, Portland, Oregon 97239; ^4^Department of Neurology, Yale School of Medicine, New Haven, Connecticut 06510; ^5^Department of Psychology, University of Oregon, Eugene, Oregon 97403; ^6^Department of Neurology, Emory University, Atlanta 30322, Georgia

**Keywords:** EEG, impulse behavior, inhibition, motor control, Parkinson’s disease, stop task

## Abstract

The ability to inhibit and adapt our behavior in response to changing stimuli is a critical component of everyday life. Individuals with Parkinson’s disease (PD) may struggle to inhibit behavior, particularly in the presence of dopaminergic therapy, which can result in impulsive behavior. Impulse control disorders are often operationalized in the laboratory using motor inhibition tasks. However, deficits of motor inhibition tasks are not always observed in PD, perhaps because of the nature of the motor inhibition that is engaged in typical tasks (e.g., suppression of incipient movement such as a button press). We employed a novel continuous movement stop task to investigate planned and unplanned motor inhibition during ongoing movement. EEG was recorded during task performance from individuals with PD (OFF and ON dopaminergic medication) and age-matched healthy controls (HC). Participants were of any sex. We found that the time it took for participants to stop a continuous movement was impaired (i.e., longer) in PD patients ON medication compared with both patients OFF medication and HC. This finding was accompanied by diminished midfrontal theta power following the stop signal in PD (ON and OFF) compared with HC. Additionally, an increase in midfrontal beta power was observed, which was higher in unplanned stopping compared with planned for all groups. However, this increase in beta occurred late—after the time of outright stopping. Together, these findings demonstrate that stopping ongoing movements was impaired in PD patients ON medication and theta and beta power play distinct roles in inhibition of movement.

## Significance Statement

Parkinson’s disease (PD) is associated with impairments in cognition and movement, both of which are engaged during motor inhibition. Using a continuous movement stop task that directly probes both functions, we tested individuals with PD (ON and OFF medications) and healthy controls using EEG. We found that levodopa medication worsened behavioral stop completion times and suppressed event-related theta (4–7 Hz) power, supporting the paradoxical nature of dopamine in cognitive control. Also, there was an increase in beta power following the stop signal; however, this increase was too late to be triggering the motor inhibition itself. Our findings demonstrate that levodopa can impair motor inhibition and support distinct roles for theta and beta activity in the termination of an ongoing movement.

## Introduction

Individuals with Parkinson’s disease (PD) often have comorbid impulse control disorders, especially in conjunction with dopaminergic medication (Zhang et al., 2008; Antonelli et al., 2011). Impulsivity is often operationalized in the laboratory with response inhibition tasks. One such task is the Stop Signal Task (SST; Logan and Cowan, 1984; Verbruggen and Logan, 2008; Lipszyc and Schachar, 2010). Here, participants must try to withhold a prepotent response (often a button press) when cued. This task has revealed diminished inhibitory control capacity in individuals with PD compared with healthy controls (Obeso et al., 2011; Swann et al., 2011; Mirabella et al., 2012).

Electrophysiological research with the SST (and similar paradigms like the Go/No-Go task) suggests that activity in beta band (13–30 Hz) is related to response inhibition (reviewed by Wessel and Anderson, 2024). Recordings from the subthalamic nucleus, part of the inhibitory control circuit, revealed an increase in beta power around the time of movement inhibition (Ray et al., 2012). This stopping-related beta increase has also been observed in cortical regions of the inhibitory control network, including sensorimotor and prefrontal cortices (Swann et al., 2009, 2011; Aron et al., 2016).

Importantly, inhibitory control is not a purely motoric phenomenon but involves cognitive elements as well, i.e., termination of motor output and recognition of the need for a change in behavior according to task demands. Further, some of the same neural networks implicated in motoric stopping are also recruited for inhibition without a motoric component—for instance, suppressing unwanted thoughts (Castiglione et al., 2019). Some studies suggest that beta band activity reflects both cognitive and motoric elements of inhibitory control (Wessel and Anderson, 2024). Theta band (4–7 Hz) activity is also observed in many cognitive tasks, including those that engage inhibitory control. For instance, increases in theta power are observed in complex conflict-related tasks that require adjustment for errors (Cavanagh and Frank, 2014; Zavala et al., 2018; Eisma et al., 2021). Though typical inhibition paradigms have been critical for exploring mechanisms of inhibitory control, their reliance on the inhibition of an incipient action presents challenges for disambiguating the cognitive and motoric components of response inhibition. That is, because identifying the need to stop (cognitive component) occurs almost contemporaneously with the implementation of movement suppression (motor component), neural signatures associated with each component are challenging to disentangle. Additionally, as these tasks elicit suppression of an intended movement (as opposed to termination of an ongoing movement), the actual time of stopping is unclear, making the precise alignment of EEG signals to the time of stopping impossible. This complication presents challenges in PD, wherein movement and cognition are impaired, but to different degrees and may be impacted differently by levodopa (Cools, 2006). Specifically, impulse control may be worsened with levodopa while movement is improved. The entanglement of these functions in the SST might explain some of the inconsistency in performance on the stop signal task in PD in relation to levodopa (Gauggel et al., 2004; George et al., 2013).

To more clearly parse the electrophysiological correlates of each component of inhibitory control, we employed a novel motor inhibition task—the Continuous Movement Stop Task (CMST; Schultz et al., 2023a,b). This task provides direct observation of motor termination on every trial. The CMST also offers the opportunity to compare stopping in different contexts—specifically when it is expected (planned) and unexpected (unplanned). These two contexts entail similar motor performance but differ in their cognitive contributions. By comparing task performance and EEG data for each condition between healthy controls, individuals with PD off dopaminergic medication, and PD patients on medication, we aim to better understand the effects of PD on the electrophysiological correlates of stopping. We hypothesized that there would be a sequential increase in theta and beta power following the “STOP” signal (with theta preceding beta), and these power increases would be greater for unplanned compared with planned conditions.

## Materials and Methods

### Participants

Twenty-five healthy age-matched controls (HC, mean age 70.4 ± 6.4, 17 F, 8 M) and 23 individuals with PD (mean age 69 ± 7.6, 8 F, 15 M) were recruited as part of a larger longitudinal study. Participants were excluded if they had another neurological disorder (aside from PD) or if they had cognitive impairment/dementia significant enough that they were unable to provide consent. All participants gave their written consent prior to the experiment and received monetary compensation for their participation. The study was approved by the Institutional Review Board of the University of Oregon (IRB 11222017.032 and 01222018.033). Data included in this manuscript were collected at their first visit. Data from follow-up visits will be published separately. Information on handedness, years of education, and cognition was collected for all groups (Table 1). For the individuals with PD, clinical ratings and information on disease duration and which (body) side had more severe symptoms were also collected (Table 1). One HC and two individuals with PD (ON medication) were excluded from EEG analysis due to noisy signals but retained for behavioral analysis (details related to criteria for EEG exclusions below). For the two participants for whom ON medication recordings were excluded due to noisy EEG, their OFF medication recordings were retained for the unpaired analyses, i.e., compared with HC, but were excluded from the paired analysis, i.e., ON versus OFF medications. This left us with 24 HC participants, 21 PD ON participants, and 23 PD OFF participants.

**Table 1.** Demographics and clinical details of participants including Montreal Cognitive Assessment (MoCA), MDS-Unified Parkinson’s disease rating Scale part III (MDS-UPDRS III)

Group	Sex	Age	Education (Y)	Handedness	MoCA	Disease duration (years)	UPDRS III (OFF meds)	UPDRS III (ON meds)
HC (*n* = 25)	17 F, 8 M	70.4 ± 6.4	17.2 ± 2.3	20 R, 2 L, 2 A	25.6 ± 2.4	-	-	-
PD (*n* = 23)	8 F, 15 M	69.4 ± 7.6	17.3 ± 3.5	21 R, 1 L, 1 A	24.3 ± 3.4	6.3 ± 6.3	42.1 ± 15.4	34.9 ± 13.4

MDS-UPDRS III was significantly worse OFF compared with ON medications (*p* = 8.086 × 10^−5^, paired *t* test, df = 22).

Given that sex was imbalanced between our HC and PD groups (*p* =0.04, Fisher’s test), we examined differences as a function of sex to rule out confounds driving our comparisons between HC and PD groups.

### Experimental protocol

Each participant performed the task twice during the same morning visit. For individuals with PD, the first session was OFF medication (12 h of dopaminergic medication abstention; PD OFF group). Testing occurred in the morning so this typically only entailed overnight abstention and patients skipping their morning medication dose. Participants arrived at the lab, rating scales were performed (detailed below), and the EEG was set up. Then EEG was acquired during the behavioral task (described below). Upon completion of the OFF EEG session, participants took their usual PD-related medications. All individuals with PD took levodopa medication, of which eight took extended-release levodopa and 17 took standard release levodopa. Additionally, six also took dopamine agonists in addition to levodopa. Testing on medication (PD ON group) occurred when patients reported medications were working or after a maximum of 1 h. EEG was recorded during task acquisition. After completing the EEG sessions, the EEG cap and electrodes were removed, and another MDS-UPDRS III rating scale (ON medications) was administered. For HC, EEG was measured in the same way in two sessions with a ∼1 h break in between (HC S1 and HC S2 group). During the 1 h break, the MoCA test was conducted for HC and individuals with PD (OFF state).

### Clinical rating scales

The MDS-Unified Parkinson’s Disease Rating Scale motor component (MDS-UPDRS III) was administered by trained research personnel and video recorded (Goetz et al., 2008). Performance on the scales was later rated by an offsite movement disorder neurologist who was blinded to the medication status of the participants (with the exception of rigidity which was scored by the research personnel). The Montreal Cognitive Assessment (MoCA) test (version 8.1) was administered by research personnel trained in standardized MoCA protocol (Nasreddine et al., 2005).

### Task

**Figure 1.**
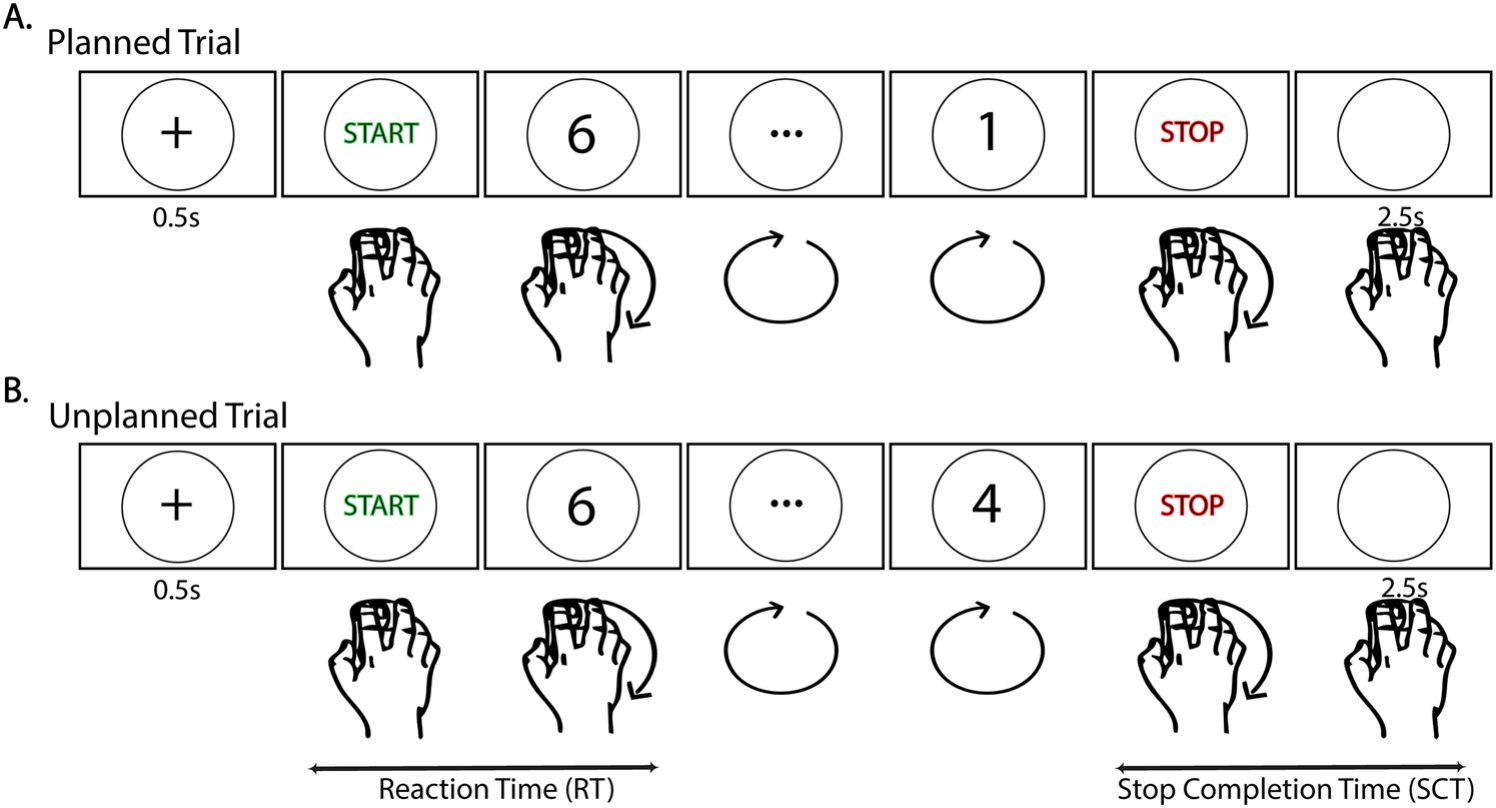
Schematic of CMST paradigm adapted from Schultz et al. (2023a). Participants were instructed to start moving a cursor controlled by a computer mouse in the clockwise direction along the circle after presentation of the “START” cue, for the duration of a countdown until a “STOP” cue was presented at which time participants were instructed to terminate their movement. For planned trials (***A***) the countdown proceeded to 1, followed by the stop signal, whereas for unplanned trials (***B***) the STOP signal occurred at a random time before 1. The countdown here for unplanned trial is shown until 4 as an example.

The CMST was adapted from previously published papers (Schultz et al., 2023a,b) and implemented in jsPsych (de Leeuw, 2015). Briefly, the task was presented on a computer screen (10.5″ × 18.5″). There were two types of trials—80 planned and 80 unplanned stop trials, which were randomly intermingled (Fig. 1). Each trial began with a presentation of 0.5 s of a fixation cue followed by a “START” cue, after which participants moved an on-screen cursor using a computer mouse with their dominant hand in a circular pattern, in the clockwise direction, at a steady pace and while a numeric countdown was simultaneously presented on the screen. The countdown began 0.75 s after “START” cue. The countdown started with a random number between 3 and 6 and decreased by 1 whole number per second. For planned stop trials, the countdown proceeded until the number “1” was displayed, after which the word “STOP” was presented 1 s later. In contrast, in an unplanned trial, the “STOP” cue appeared at a random (unpredictable) time before the countdown reached 1. Participants were told they should stop moving upon seeing the “STOP” signal and that on most trials the countdown would proceed all the way to 1 but on some trials the “STOP” signal might occur early. After the “STOP” cue, there was 2.5 s interval for response termination and intertrial interval prior to the next fixation presentation.

All participants received initial training to understand the task better. During this training, participants were instructed to move in a clockwise direction and got feedback after every trial if they moved in the wrong direction (i.e., anticlockwise). Feedback was also provided if they were too slow (moving the mouse less than one pixel between successive cursor samples, i.e., the Euclidean distance), they stopped early (i.e., if they stopped before the “STOP” cue), they were too slow to stop (movement was terminated >500 ms after the “STOP” cue), or if their computer mouse movements deviated far from the circle outline (>50 pixels from the circle). This feedback was provided during training only—not during the actual task presentation.

### EEG data recording

We recorded scalp EEG data using the ActiveTwo Biosemi-64 channel system at a sampling rate of 1,024 Hz. EEG electrodes were placed according to a standard 10–20 montage. An additional four external (flat) electrodes were placed laterally to each eye and above and below one eye to detect ocular artifacts. Electromyogram (EMG) was recorded from the dominant arm (approximate flexor carpi ulnaris muscle) for HC. For individuals with PD, EMG was recorded from the arm (approximately the flexor carpi ulnaris muscle) and the leg (approximately the gastrocnemius muscle) on the side corresponding to the most severe symptoms. EMG was only used to verify stop and start periods of tremor, should it occur. The task was synchronized to the EEG data acquisition device using the built-in Biosemi triggering cable.

Additionally, a photodiode (custom Biosemi) was also placed in the lower corner of the computer screen to record light changes corresponding to stimulus presentations on the screen directly. This photodiode signal was digitized with the EEG data during acquisition and used to confirm event onsets. These photodiode deflections were used to determine event times for EEG analysis.

### Behavioral analysis

The “START” reaction time (RT) was calculated as the time between the presentation of the “START” cue and the onset of the computer mouse movement. The stop completion time (SCT) was measured as the time between the presentation of the “STOP” cue and when the computer mouse came to full arrest. Trials were excluded from both behavioral and EEG analysis based on the following criteria: (1) RTs longer than 1.5 s, (2) cursor was moved in the incorrect (counterclockwise) direction, (3) cursor stopped before the presentation of the “STOP” cue, and (4) no movement was initiated. After removing incorrect trials, the mean RT and SCT (separately for planned and unplanned trials) were determined for every subject. Note that one subject performed most trials in the counterclockwise direction. Because we interpreted this to be a misunderstanding in the instructions and performance on the task was otherwise correct, we included this subject in the analysis and included all trials clockwise and counterclockwise. However, excluding this subject did not alter the results.

### EEG data preprocessing

EEG data were preprocessed with the MNE Python toolbox (Gramfort et al., 2014), using the following steps: (1) power line noise (60, 120, 180, 240 Hz) was removed using the *mne.filter.notch_filter* and a “biosemi64” channel montage was assigned; (2) data was high-pass filtered at 1 Hz using *mne.filter.filter_data*; (3) periods that were strongly contaminated with large muscle artifacts as well as consistently noisy channels were identified via visual inspection; (4) noisy channels were interpolated using *interpolate_bads* (typically only 1–2 channels per file was interpolated with a maximum of four channels); (5) data was rereferenced to an average reference; (6) ICA (“*fastica*”) was performed and components corresponding to ocular artifacts were removed; (7) a noise rejection threshold was applied using peak threshold of above 300 × 10^−6^ and below 1 × 10^−6 ^μV when creating event-related epochs. After applying this threshold, an average of 72 trials were retained across all participants for each condition, (8) EEG signals were epoched and aligned to the “START” cue, “START” response (i.e., movement initiation), the “STOP” cue, or the time of “STOP” response (i.e., the time of movement cessation). In each case, the epochs began 1.5 s before and ended 1.5 s after the relevant event. For epochs aligned to the “STOP” cue and movement termination (“STOP” response), separate epochs were created for planned and unplanned stop trials and (9) data were downsampled to 512 Hz for faster computation.

### EEG time–frequency analysis

To obtain time–frequency data for each trial, “morlet wavelets” (*mne.time_frequency.tfr_morlet*) were used for frequencies ranging from 2 to 50 Hz with a step of 1 and the number of cycles set to “frequency/2.” The time–frequency data was further decimated by a factor of 4 for faster computation. Trials were averaged, and an average (across both planned and unplanned trials) baseline period correction was applied on averaged trials using the *z*-score method. The baseline was defined as 500 ms during the intertrial interval, prior to the presentation of the fixation indicating the beginning of the trial. Further, the epochs were cropped to −0.5 to 1 s relative to each event. For each participant, theta (4–7 Hz) and beta (13–30 Hz) power were extracted separately by averaging the power across the corresponding frequencies for the midfrontal (averaged from FCz and Fz electrodes), left sensorimotor (C3 electrode), and right sensorimotor (C4 electrode) regions. We initially had also planned an analysis of lateral frontal channels (lateral right, average of “FC6,” “F6,” “F8,” and left frontal, average of “FC5,” “F5,” “F7”), given the role of these regions in inhibition of movement. However, this analysis was abandoned due to excessive residual muscle artifacts in these channels which required interpolation.

### Statistical analysis

#### Demographic statistics

Welch’s paired *t-*tests were used to determine significant differences between groups for continuous variables and Fisher’s test to assess if there were any differences for the “sex” variable between groups.

#### Behavioral statistics

We used a linear mixed effects model (lme4, version 1.1.34; Bates et al., 2015) to determine differences in RT and SCT separately across groups (HC S1, HC S2, PD OFF and PD ON groups) and conditions (planned and unplanned trials) using R (R Core Team, 2023). We regressed SCT onto the primary variables of interest, group, and condition:

$$\begin{array}{rl} & \mathrm{SCT}=\beta_{0i}+\beta_{1}\mathrm{Group}+\beta_{2}\mathrm{Condition}+\beta_{3}(\mathrm{Group}\times \mathrm{Condition})+\beta_{4}\mathrm{Sex}\\ & \beta_{0i}=\gamma_{00}+\theta_{i}, \end{array}$$

in which intercepts are allowed to vary across participants (the variability of which is indexed by *θ_i_*). Similarly, we regressed RT onto only group:

$$\begin{array}{rl} & \mathrm{RT}=\beta_{0i}+\beta_{1}\mathrm{Group}+\beta_{2}\mathrm{Sex}\\ & \beta_{0i}=\gamma_{00}+\theta_{i}. \end{array}$$

Note that participant sex is included in both models to test whether sex was associated with outcomes above and beyond the impact of group and condition. We performed model-implied post hoc comparisons across groups (e.g., HC S1 to PD ON), conditions (planned to unplanned), and unique group–condition combinations, using a false discovery rate (FDR) correction to minimize Type I error. Paired comparisons were performed for all within-group comparisons (i.e., unplanned vs planned) and also for comparisons between medication status (PD ON vs OFF and HC S1 vs HC S2). Unpaired tests were used to compare patients to HC (HC S1 vs PD OFF and HC S2 and PD ON).

To determine differences between behavioral performance accuracy between groups, Mann–Whitney unpaired *t* tests were used. Also, to measure variability of SCT, a Levene’s test followed by coefficient of variation (CV) was performed. A bootstrap with replacement (10,000 times) was implemented to determine statistical differences between groups and conditions, followed by FDR correction for multiple comparisons.

#### Electrophysiology statistics

A cluster-level statistical permutation test was conducted to compare across groups and conditions for average theta and beta power determined 0.5 s before and 1 s after “START” and “STOP” cue/response. The MNE statistics functions *mne.stats.permutation_cluster_test* and *mne.stats.permutation_cluster_1samp_test* were used for unpaired and paired tests, respectively (same groups comparisons as described above). In this test: (1) Along the time axis, clusters were grouped if the consecutive time points with a test statistic value exceeded *p* < 0.05. (2) In every cluster, cluster-level statistics were obtained from the sum of *t* values. (3) The group (HC or PD OFF or PD ON) or condition label (planned or unplanned) was shuffled randomly (10,000 permutations) to obtain a null distribution. From each shuffle, the highest cluster statistic value was noted. (4) Last, to determine a significant cluster, the cluster-level statistical value had to be above the 95th percentile of the null distribution (hence correcting multiple comparisons for time axis and accounting familywise error rate; Maris and Oostenveld, 2007).

Next, to determine if the peak time of theta and beta power differed across time for the “STOP” cue, a mixed effects model (lmer) was used to identify both within conditions and across groups (within and between groups) and frequency (theta or beta band). We regressed peak times onto the primary variables of interest, group, condition, and frequency:

$$\begin{array}{rl} & \mathrm{PeakTime}=\beta 0+\beta 1\mathrm{Group}+\beta 2\mathrm{Condition}+\beta 3\mathrm{Frequency}+\beta 4(\mathrm{Group}\times \mathrm{Condition})\\ & +\beta 5(\mathrm{Group}\times \mathrm{Frequency})+\beta 6(\mathrm{Condition}\times \mathrm{Frequency})+\beta 7(\mathrm{Group}\times \mathrm{Condition}\times \mathrm{Frequency})+\beta 8\mathrm{Sex}\\ & \beta_{0i}=\gamma_{00}+\theta_{i}, \end{array}$$

where each subject was a random intercept, followed by post hoc FDR correction for multiple comparisons for paired and unpaired groups or conditions.

## Results

### Clinical and behavioral results

The MDS-UPDRS III scores were significantly greater (indicating worse impairment) OFF medication compared with ON medication in individuals with PD (*p* = 8.086 × 10^−5^; Table 1). This suggests medications reduced motor symptoms. The MoCA scores did not differ significantly between groups. All groups performed the task with high accuracy (93% for patients OFF, 93% for patients ON, and 99% for HC S1 and 98% for HC S2; Table 1). But the accuracy was significantly lower in PD OFF compared with HC S1 groups (*p* = 0.0007, unpaired *t* test).

**Figure 2.**
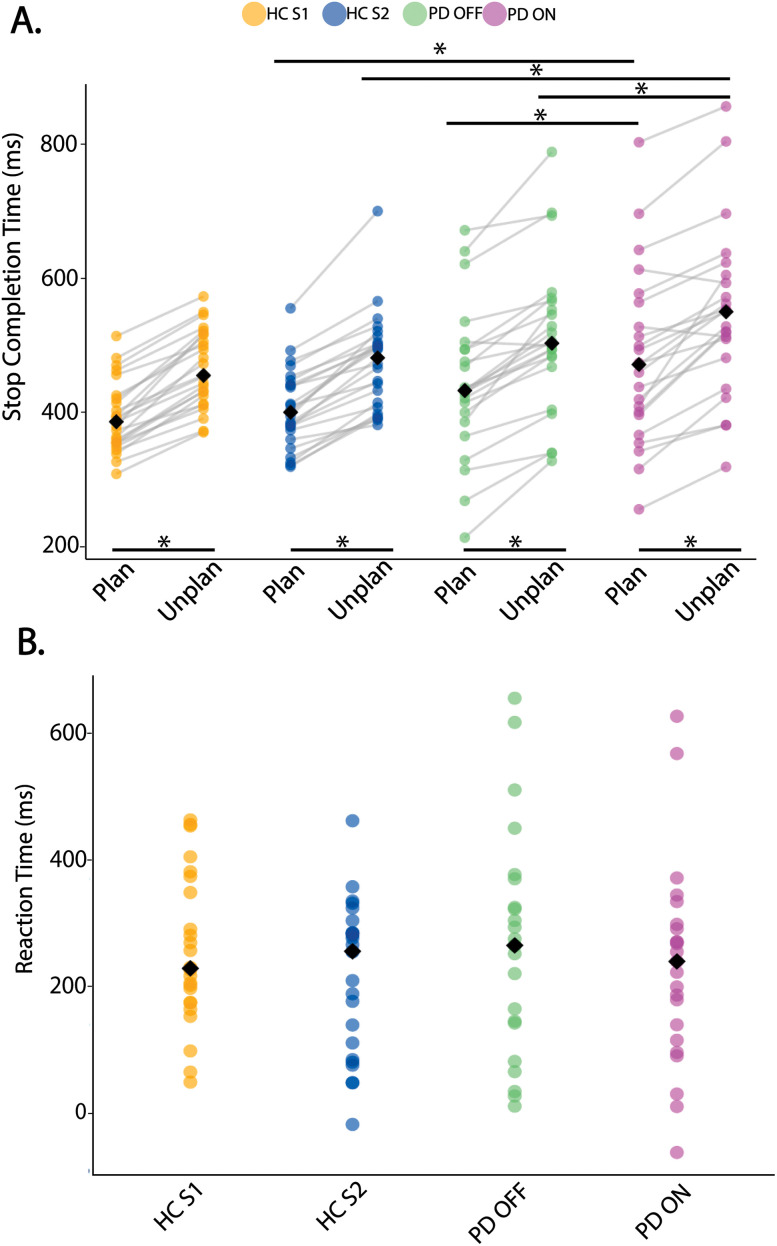
Behavioral results. ***A***, Stop completion times (SCT) for planned and unplanned trials across groups. Unplanned SCTs were longer (i.e., slower stopping) compared with planned SCTs for all groups. Also, SCTs in PD ON were longer than HC and PD OFF for both types of trials. ***B***, Reaction times (RT) across groups. There were no significant differences across groups for RT. Solid lines with asterisks indicate significance between and within groups (median indicated by black diamond, FDR corrected significance *p < 0.05; see Extended Data Fig. 2-1 for exact *p* values). CV values and their associated *p* values for these measures are included in Extended Data Figures 2-2, 2-3, respectively.

We observed that SCT was significantly longer for unplanned than planned trials, and this difference was present for all groups (*p* < 0.05, FDR corrected; Fig. 2*A*). Next, between-group comparisons revealed that SCT was significantly higher (indicating stopping was slower) in PD ON compared with HC S2 and PD OFF, for both planned and unplanned trials (*p* < 0.05, FDR corrected, see Extended Data Fig. 2-1 for exact *p* values). There were no significant differences in RT across groups.

10.1523/ENEURO.0286-25.2025.f2-1 Figure 2-1 **Table showing p-values for comparison between groups and conditions for SCT.** Asterisk denotes significance (p < 0.05, FDR corrected). Download Figure 2-1, DOCX file.

10.1523/ENEURO.0286-25.2025.f2-2 Figure 2-2 **Coefficient of variation (CV) across groups and conditions for SCT**. Black lines with an asterisk denote significance (p < 0.05, FDR corrected). The SCT was more variable for PD OFF than HC S1 groups for SCT for both planned and unplanned stopping. Download Figure 2-2, TIF file.

**Figure 3.**
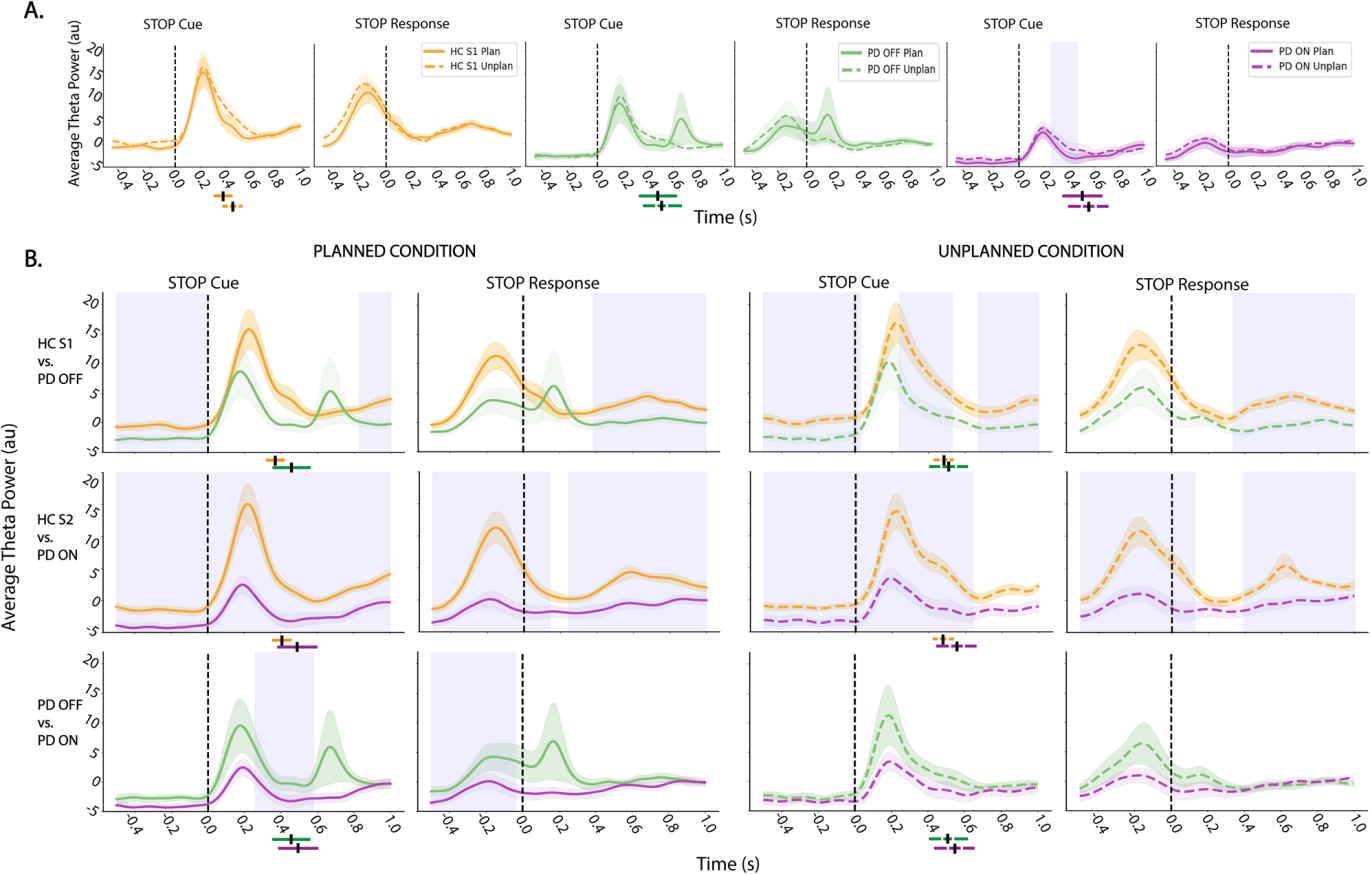
Average theta power in midfrontal cortex. ***A***, Average theta (4–7 Hz) power for within-group comparison of planned stopping (solid line) to unplanned stopping (dotted line), time locked to both the stop cue (“STOP Cue,” left) and time of actual motor cessation (“STOP response,” right). ***B***, Average theta power comparison between group comparisons for planned stop trials (left 2 columns) and unplanned stop trials (right two columns), time locked to both the stop cue (“STOP cue”) and time of motor cessation (“STOP response”). The top row compares the HC S1 with PD OFF medication group, the middle row compares HC S2 with PD ON medication group, and the bottom row compares PD OFF with ON medication group. For each analysis, time zero is indicated with a dashed line. Shaded regions indicate time periods that are significant (*p* < 0.05, cluster-based correction for multiple comparisons). The shaded portion around each power trace represents standard error of the mean (SEM). The mean SCT is denoted with a black line below the plot for each group and condition and the standard deviation for the respective group or condition is indicated with a colored bar around the black line. Most of the differences were between groups with theta power being highest for HCs and lowest for patients ON medication. See Extended Data Figure 3-1 for a control analysis to verify these between-group differences were not driven by sex imbalances between groups.

10.1523/ENEURO.0286-25.2025.f2-3 Figure 2-3 **Table showing p-values for group and condition comparisons of CV for SCT**. Asterisk denotes significance (p < 0.05, FDR corrected). Download Figure 2-3, DOCX file.

Next, a Levene’s test revealed a significant difference in SCT variance across groups (*p* = 6.282 × 10^−4^), with CV being higher for PD OFF compared with HC (*p* < 0.05; Extended Data Figs. 2-2, 2-3).

Finally, because our groups were not balanced for sex, sex was included as a covariate in a linear mixed effect model assessing for SCT and RT and no significant effects were observed due to “Sex.” Thus, behavioral results were likely not impacted by sex.

### Electrophysiology spectral power results

#### Stopping results

##### Midfrontal theta power

The STOP cue was followed by an increase in theta power for all groups. For the PD ON group, there was greater theta power for unplanned stop trials compared with planned stop trials when aligned to the “STOP” cue (*p* < 0.05, cluster-based correction); however, this effect was not seen for other groups or when events were aligned to the time of “STOP” response (i.e., time of movement cessation; Fig. 3*A*). For between-group comparisons (Fig. 3*B*), and both planned and unplanned stopping, there were numerous periods of time both before and after the “STOP” cue, with significant differences between the HC S1 and PD OFF medication groups. Specifically, theta power was higher in HC S1 than the PD OFF medication group (*p* < 0.05, cluster-based correction). A similar pattern was present for the HC S2 compared with the PD ON medication groups. In fact, theta power was significantly higher for the HC S2 group compared with the PD ON group for most of the time before and after the “STOP” cue, again for both planned and unplanned stop trials (*p* < 0.05, cluster-based correction). Finally for the PD OFF compared with the PD ON medication group, there were time periods where theta power was significantly higher in the PD OFF compared with PD ON medication groups for planned stop trials aligned to both the stop cue or stop response (*p* < 0.05, cluster-based correction). However, there were no differences between the groups for unplanned stop trials. Overall, theta power was higher in HC than PD groups before and after both the “STOP” cue and response, irrespective of condition, and theta power was higher for PD OFF compared with ON for certain time intervals for planned stop trials only.

10.1523/ENEURO.0286-25.2025.f3-1 Figure 3-1 **Average theta power in midfrontal cortex comparing between males and females across groups**. Time-locked to both the stop cue (“STOP Cue”, left) and time of actual motor cessation (“STOP response”, right)**.** Theta traces comparing male and females across HC S1 and PD OFF groups for planned (A) and unplanned (B) conditions. Theta trace comparing male and females across HC S2 and PD ON groups for planned (C) and unplanned (D) conditions. For each analysis, time zero is indicated with a dashed line. Light blue shaded regions show time periods that are significant (p < 0.05, cluster-based time correction for multiple comparison). The shaded area region surrounding each power trace represents standard error. Theta differences between males and females exist, but compared to the HC S1 and PD OFF groups the differences persist over shorter time windows and mostly occur well after the cue or response. Download Figure 3-1, TIF file.

##### Midfrontal beta power

**Figure 4.**
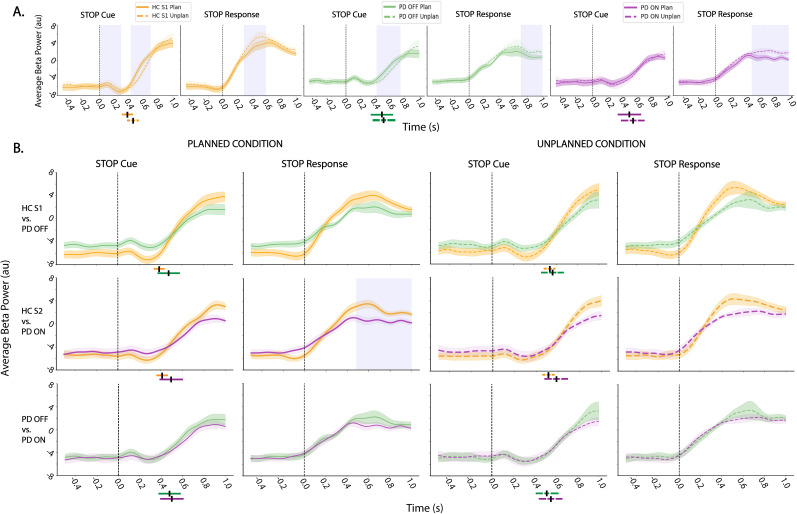
Average beta power in midfrontal cortex. Same as Figure 3, but depicting beta (13–30 Hz) activity. ***A***, Average beta power for within-group comparison of planned stopping (solid line) to unplanned stopping (dotted line), time locked to both the stop cue (“STOP Cue,” left) and time of actual motor cessation (“STOP response,” right). ***B***, Average beta power comparison between groups for planned stop trials (left two columns) and unplanned stop trials (right two columns), time locked to both the stop cue (“STOP cue”) and time of motor cessation (“STOP response”). The top row compares the HC S1 with the PD OFF medication group, the middle row compares HC S2 with the PD ON medication group, and the bottom row compares PD OFF with ON medication. For each analysis, time zero is indicated with a dashed line. Blue shaded regions indicate significant time periods (*p* < 0.05, cluster-based correction for multiple comparisons). The shaded area around each power trace represents SEM. The mean SCT is denoted with a black line below the plots for each group and condition and the standard deviation for the respective group or condition is indicated with a colored bar around the black line. Most of the differences are within groups with beta power being earlier to rise for planned stop trials (likely due to faster stopping), but reaching an overall higher peak for unplanned stopping.

Beta power (Fig. 4*A*) was higher for the planned compared with unplanned stopping following the “STOP” cue for the HC and PD OFF groups (*p* < 0.05, cluster-based correction), but not for PD ON. However, when aligned to “STOP” response, beta for unplanned stopping was higher than planned stopping after movement termination for all groups. This likely reflects an overall pattern of beta power rising with motor termination, and this occurring earlier for planned stop trials (consistent with faster SCT) but reaching an overall higher peak for unplanned stop trials. Additionally, for the HC S1 group, beta power for unplanned stop trials was higher than planned beta power shortly after the “STOP” cue for a brief time period. Next, for between-group comparisons (Fig. 4*B*), the only significant difference was that beta power was significantly higher in the HC S2 compared with the PD ON medication group for the planned stop conditions following the “STOP” response. Notably, beta power peaked well after the cessation of motor activity. Overall, unlike theta, beta power was less different between groups, but more impacted by trial type.

##### Sensorimotor activity: theta and beta

**Figure 5.**
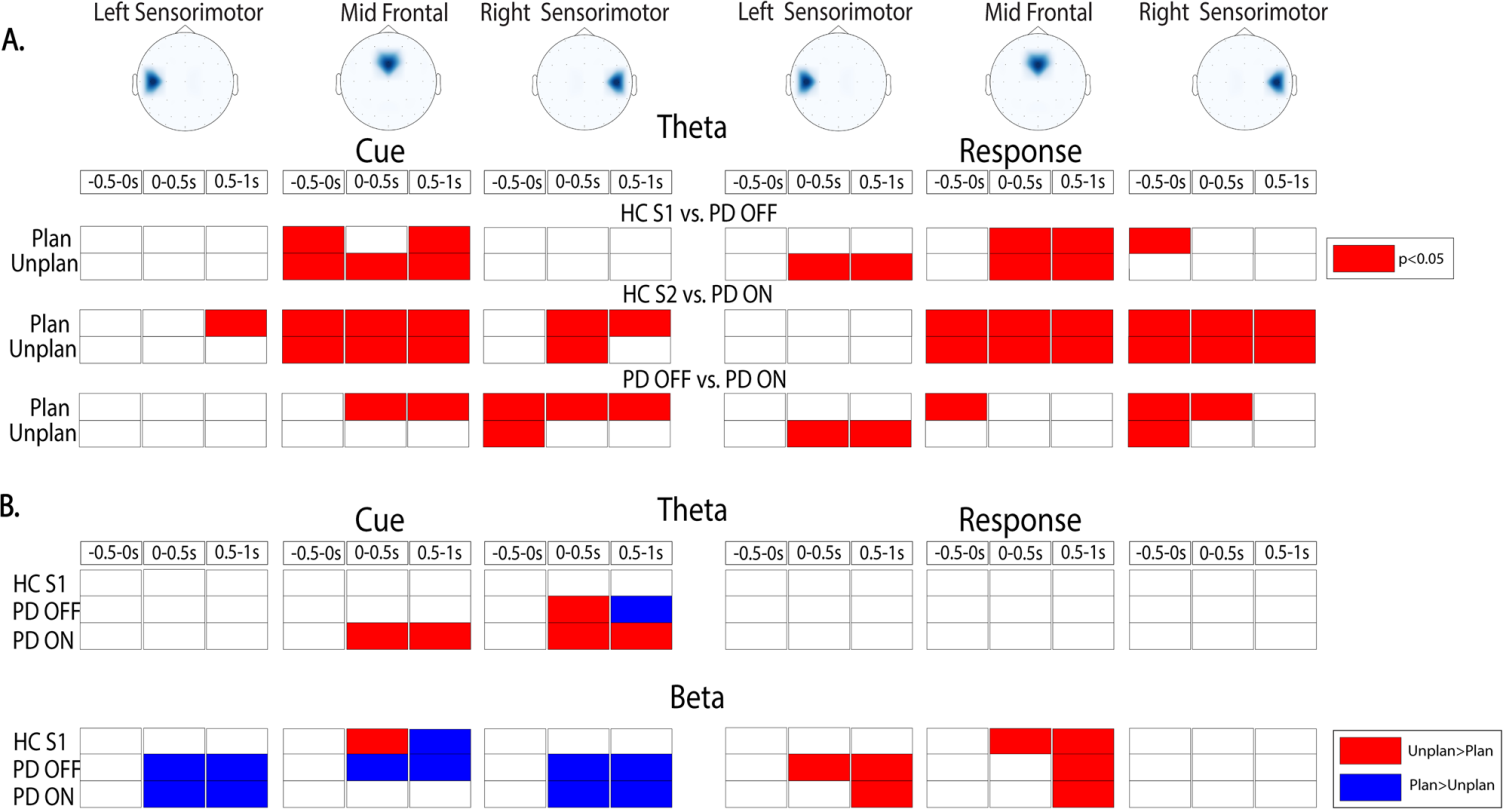
Summary EEG results for stopping. ***A***, Between-group comparisons. Significant average power for planned and unplanned conditions, across time: −0.5 to 0, 0–0.5, and 0.5–1 s relative to event onset. Time zero is aligned to either the stop cue (“CUE,” left) or the time of motor cessation (“STOP” response, right) for left sensorimotor, midfrontal, and right sensorimotor cortex. Red cells indicate significance (*p* < 0.05, cluster-based time correction for multiple comparison) where power is higher in HCs (top two rows) or PD OFF (bottom row). Each region of interest is depicted above the matrix, with its name displayed at the top. Note that beta power is not shown because only one cell (which was higher in HC S2 than PD ON for planned stop trials only after movement cessation) was significant. ***B***, Within-group comparisons. Significant average theta (top) and beta (bottom) power for planned versus unplanned conditions, across time (−0.5 to 0 s before and 0–0.5 s and 0.5–1 s of event onset) aligned to either the stop cue (“CUE,” left) or the time of motor cessation (“STOP” response, right) for within-group comparisons over left sensorimotor, midfrontal, and right sensorimotor cortices. Red cells indicate significance when unplanned stopping is greater than planned stopped condition and blue cells indicate the opposite.

We also examined theta and beta power over right and left sensorimotor regions between and within groups. Here, we saw similar patterns of theta and beta power changes as was observed in midfrontal regions—particularly for the right (ipsilateral to movement), but less so for the left (contralateral to movement), sensorimotor cortex. An overall overview of theta and beta patterns for stopping behavior for all ROIs is presented in Figure 5.

##### Control for sex

Because the sex distribution of our groups differed, we tested whether our primary results indicating differences between patients and controls (i.e., theta power in stopping-conditions) were more strongly modulated when dividing groups by sex rather than disease state (i.e., grouping as male vs female as opposed to PD vs HC). In this analysis, results were less apparent than when grouping by disease state. Specifically, the window of significance was limited and occurred well after the STOP cue and also the SCT (Extended Data Fig. 3-1). Thus although there were some time windows which were significantly different, they were fewer than when grouping by disease state, and in all cases, they occurred much later after the event onset (“STOP” cue or response). Hence, it is most likely that disease state (i.e., PD vs HC) and not sex contributed most to our observed findings.

##### Timing of theta relative to beta

**Figure 6.**
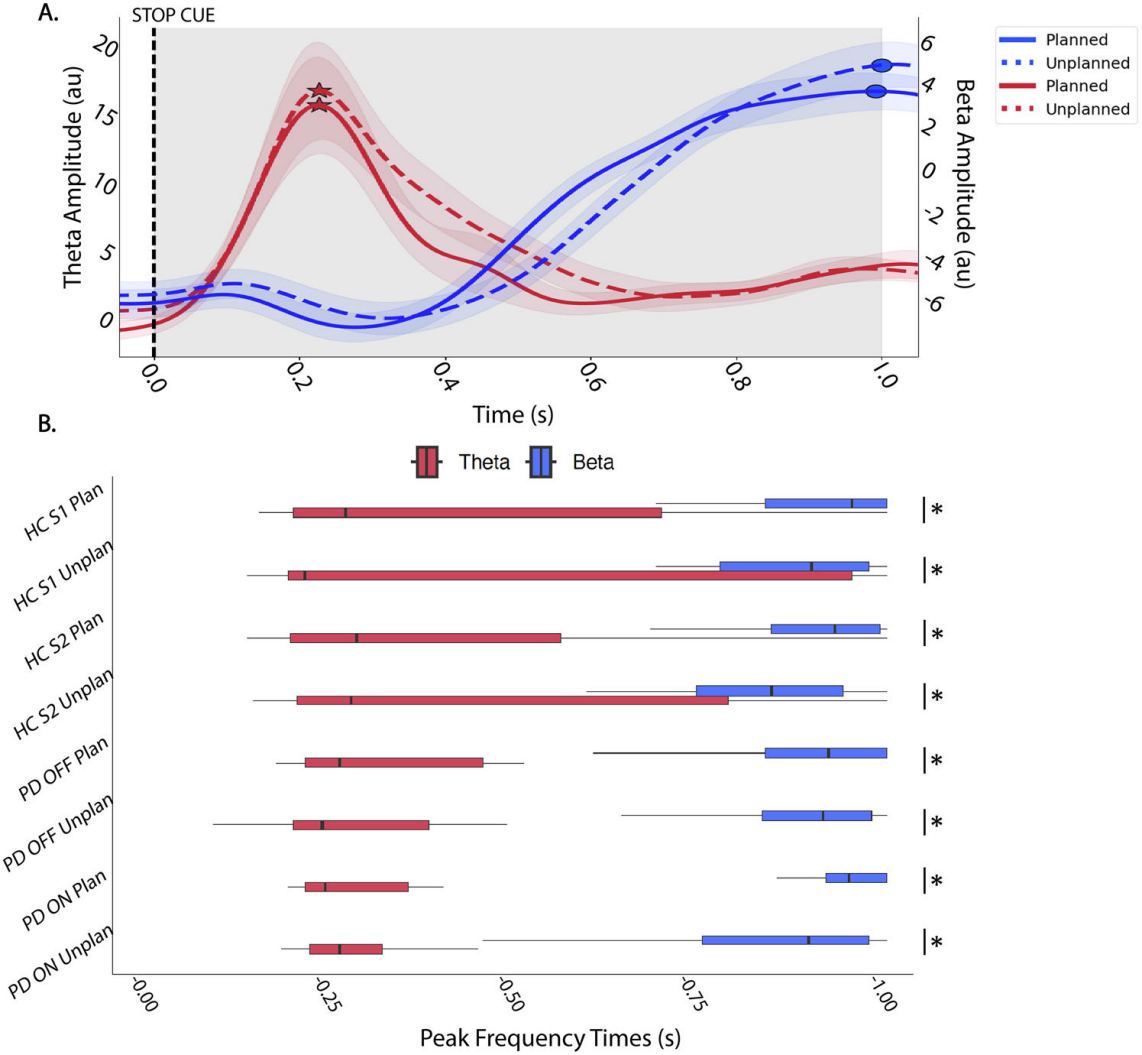
Time of peak theta and beta following the stop cue. ***A***, Average theta and beta for HC S1, demonstrating the derivation of the time at which the peak in theta (red) and beta (blue) power occurs in the 1 s window (gray shaded area) following the stop cue (peak power indicated by stars and circles for theta and beta, respectively). Solid lines indicated planned stop trials and dotted lines indicated unplanned stop trials. Shaded regions around each power trace represent SEM. ***B***, Boxplots showing time at which peak theta (red) and beta (blue) occurred across groups and conditions. Black lines with asterisks indicate significance (*p* < 0.05, FDR corrected; see Extended Data Fig. 6-1 for exact *p* values). Theta power peaked significantly earlier than beta power for each group.

We also examined the latency differences for theta and beta power fluctuations. We calculated the time when averaged theta and beta power peaked after the “STOP” cue (Fig. 6*A*). The theta peak latency was significantly earlier than beta peak latency (*p* < 0.05, FDR corrected) within each group and for both trial types (Fig. 6*B*, Extended Data Fig. 6-1 for exact *p* values).

10.1523/ENEURO.0286-25.2025.f6-1 Figure 6-1 **Table showing p-values for comparison of theta and beta peak for each group and condition**. Asterisk denotes significance (p < 0.05, FDR corrected). Download Figure 6-1, DOCX file.

#### Movement onset results

**Figure 7.**
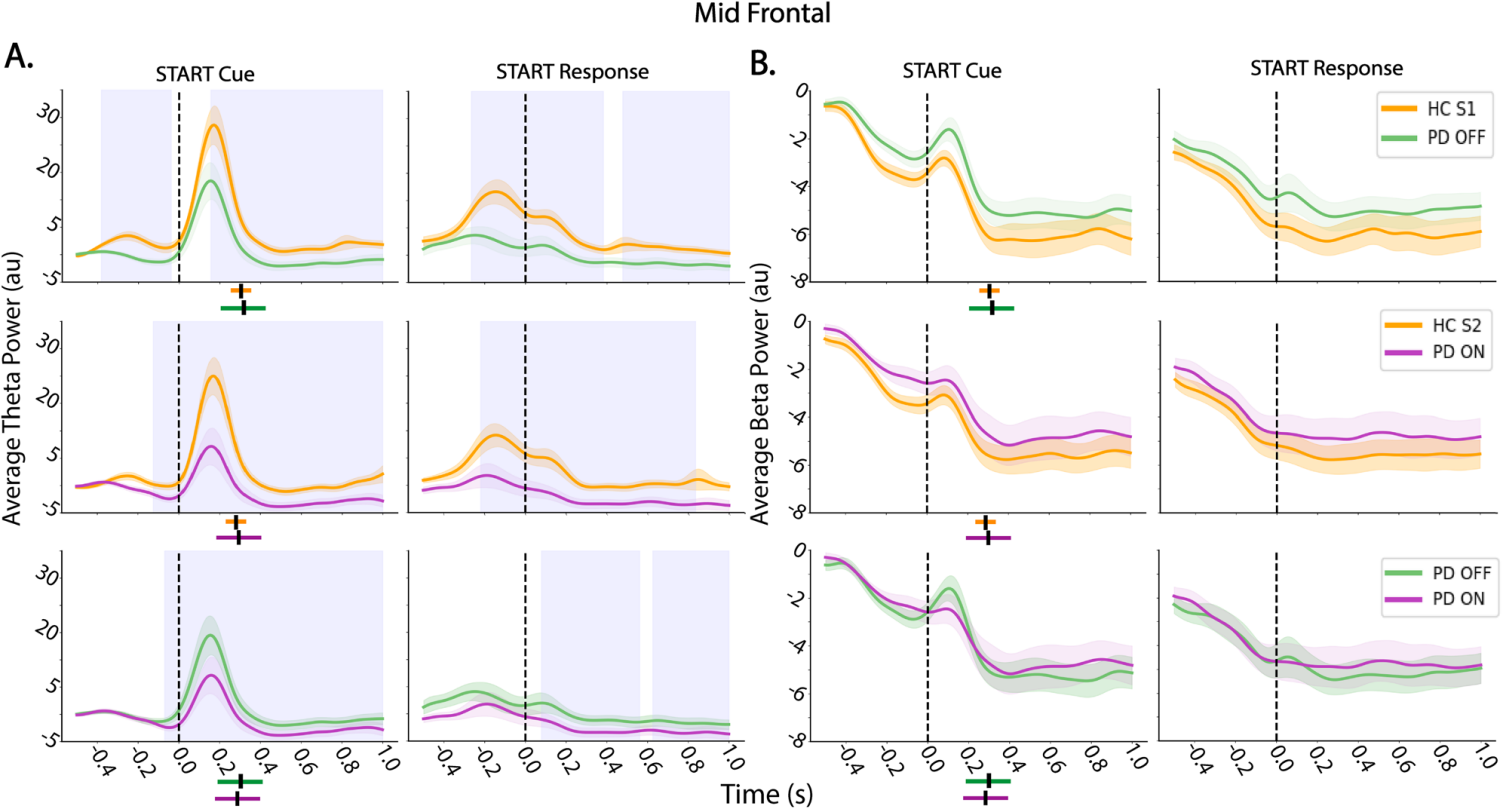
Average theta and beta power for movement initiation. ***A***, Comparison of midfrontal theta power between groups aligned to the start cue (“START” cue) and movement initiation (“START” response). ***B***, Comparison of midfrontal beta power between groups aligned to the start cue (“START” cue) and movement initiation (“START” response). For each analysis, time zero is indicated with a dashed line. Blue shaded regions indicate significant time periods (*p* < 0.05, cluster-based time correction for multiple comparisons). The shaded area surrounding each power trace represents SEM. The mean RT is denoted with a black line below the plots for each group and condition, and standard deviation for the respective group or condition is indicated with a colored bar around the black line. There were between-group differences following the go cue for theta but not beta. For sensorimotor cortex results, see Extended Data Figure 7-1.

Given theta and beta power changes were observed for “STOP” cue and response, an exploratory analysis was conducted aligned to the “START” cue and RT (i.e., “START response”). For the between-group comparisons (Fig. 7*A*), midfrontal theta power increased after the “START” cue and was significantly greater for HC S1 compared with PD OFF and HC S2 compared with PD ON for time windows before and after the “START” cue and time of movement initiation. Further, theta was significantly greater in PD OFF than PD ON medication for the “START” cue and response but primarily after the event onset. We also examined beta power for the “START” cue and response (Fig. 7*B*). As expected in the context of movement over sensorimotor regions (Pfurtscheller, 1981), beta power decreased shortly after the “START” cue (after a brief increase) and further decreased following movement initiation (and a similar pattern was observed in sensorimotor regions). However, there were no significant differences between groups for beta power. Overall, midfrontal theta, but not beta power, was modulated between groups at the time of movement initiation such that theta power was higher in HC compared with patients and in the PD OFF compared with ON medication group (Fig. 7*B*, Extended Data Fig. 7-1 for data over sensorimotor regions).

10.1523/ENEURO.0286-25.2025.f7-1 Figure 7-1 **Average theta and beta power for movement initiation in left sensorimotor cortex**. Average theta power (top row) and beta power (bottom row) compared across groups aligned to start cue. For each analysis, time zero is indicated with a dashed line. Blue shaded regions show time periods that are significant (p < 0.05, cluster- based time correction for multiple comparison). The shaded area region surrounding each power trace represents standard error. Overall theta power in the left sensorimotor region is similar to the midfrontal region, but of lower amplitude than the midfrontal cortex for all groups. Sensorimotor beta power decreases following the signal to move, as expected (Pfurtscheller, 1981), but does not differ between groups. Download Figure 7-1, TIF file.

## Discussion

We used a novel task to study stopping a continuous movement in people with PD (ON and OFF medications) and HC. We found that stopping was slower in individuals with PD ON medications compared with OFF medications and HC. Next, we observed that event-related theta power was diminished in individuals with PD compared with HC groups and that theta power was lower still in PD ON compared with OFF medications. Finally, we found that beta power after the stop signal was higher in unplanned compared with planned stopping for all groups but that this effect was late—mainly occurring after the time of stopping.

### Levodopa impaired behavioral stopping

Prior work in young control participants observed that unplanned stopping took longer than planned (Schultz et al., 2023a,b). Here we demonstrated this effect was retained in older controls as well as people with PD both ON and OFF medications. Additionally, PD patients took longer to stop ON compared with OFF medication. This finding of worsened performance ON medication aligns with theories suggesting that an “overdosing” of dopamine could negatively impact cognition (Cools, 2006), possibly contributing to impulse control disorders sometimes observed in PD (Zhang et al., 2008; Antonelli et al., 2011). It is hypothesized that in early stages of disease, levodopa could act as an “overdose” of dopamine in brain areas that are relatively spared from early degeneration such as prefrontal cortex and ventral striatum. This overdose can result in cognitive impairment (Vaillancourt et al., 2013). Dopamine agonists can also contribute to impulsivity in PD (Vaillancourt et al., 2013; Carbone and Djamshidian, 2024). Given that in our cohort 26% of the patients took dopamine agonists and 61% of patients had a disease duration of less than 5 years, either dopamine overdose (earlier in disease), dopamine agonists, or a combination of the two, may explain the impaired stopping observed ON medication. Finally, SCT was more variable in patients OFF medication compared with HC. The increased variability could reflect the variance in the disease severity. A non-mutually exclusive explanation may be that patients are more likely to use different strategies on different trials compared with HC.

### Theta was modulated by task-relevant stimuli and differed across groups

Examination of task-related electrophysiology revealed midfrontal theta power increased following START and STOP cues which were diminished in the PD compared with the HC group. This observation aligns with previous work demonstrating an attenuated midfrontal theta response associated with impaired cognition in PD (Singh et al., 2018). Given that theta power modulation during our task appeared to be associated with cue presentation in general (i.e., it was present following both “START” and “STOP” cues), it is possible that diminished midfrontal theta power during the CMST reflects disruptions of attention or monitoring. This interpretation is supported by previous work, which suggests that midfrontal theta power reflects activation of a cortico-STN pathway indicating a need for control which triggers motoric updates implemented by the STN (Zavala et al., 2018; Choi et al., 2024).

In addition to the diminished theta power observed in PD compared with HC, within the PD group there was also reduced theta power in ON compared with OFF medication. This parallels the behavioral deficit in SCT we observed in the ON condition and suggest that theta power is further modulated by dopamine and may be altered by a dopamine “overdose” as described in the prior section.

### Late beta was elevated for unplanned stopping in all groups

Analysis of beta oscillations revealed that midfrontal beta power rose following the STOP response (i.e., outright stopping) and that this rise was higher for unplanned compared with planned stop trials for all three groups. Because the CMST task allows locking directly to the time of outright stopping, we showed that the largest increase in beta power occurs after motor termination—too late to be causal for the stopping. This finding argues against the proposed role of beta in implementing stopping (Swann et al., 2009; Aron et al., 2016; Schmidt et al., 2019). One possibility is that this relatively late increase in beta power may reflect the “clear out” function ascribed to beta, where information is cleared out or reset before the next trial (Schmidt et al., 2019). Additionally, our observation that this late beta power increase was higher for unplanned stop trials could indicate that greater cognitive engagement is required for this “clear out” after an unanticipated stop. Another interpretation (not necessarily mutually exclusive) is that the late increase in beta may reflect increased engagement of cognitive control following unplanned stop trials which may manifest as increased caution for the subsequent trial. This may be akin to the modulations in beta which have been observed following errors (Koelewijn et al., 2008; Tan et al., 2014a,b; Torrecillos et al., 2015; Zavala et al., 2018; Patai et al., 2022). For future analysis, it would be interesting to explore this phenomenon where the trial types can be divided based on prior trial, that is, post response beta power for a trial preceded by an unplanned versus planned stop.

While the largest beta increase was too late to trigger motor inhibition, we did observe a small increase in beta power after the STOP cue but before full motor arrest in all groups. It is possible that this more subtle increase could have been playing a role in motor inhibition and that it is perhaps just dwarfed in EEG by the prominent beta rebound after movement, well known to occur in sensorimotor regions and volume conducted to other electrodes (Pfurtscheller, 1981). One way to interpret this cascade of signals is in the context of the multistage *Pause-Retune-then-Cancel* model of action stopping (Schmidt and Berke, 2017; Diesburg and Wessel, 2021; Hervault and Wessel, 2025). This model suggests that action stopping may begin with a “pause” component that reflects initial attention-related cue identification processes, then a “retune” period to calibrate the adjustment of movement needed to complete the trial and, in the context of a stopping task—a “cancel” component wherein movement is terminated (Hervault and Wessel, 2025). In this context, the small early beta increase could reflect the “pause” process, the theta effect may be part of the retune process, and the later, very large beta may relate to the “cancel” process, wherein movement termination is retained.

### Limitations

Several limitations of the current study should be considered. For example, we were unable to balance the order in which the PD group performed the CMST. That is, all patients first performed the task while off, then while on medication. We controlled for possible order effects by also having the HC group perform the task twice, but residual order effects cannot be entirely ruled out. Additionally, some of the PD patients showed minimal improvements following administration of medication, as demonstrated by negligible changes in their MDS-UPDRS III scores. This heterogeneity in medication response could obscure differences when averaging across subjects. Because of possible disparate effects on motor and cognitive symptoms (perhaps due to asymmetry in manifestations of neurodegenerative patterns; Cools, 2006), changes in motor symptoms may not be reflective of the impact of levodopa on cognition. As such, we opted to include all participants (even those who did not exhibit a meaningful clinical improvement after taking medication) since they were still tested in the context of levodopa. Finally, much of the PD group presented with resting state tremor, which could have affected task performance as there is constant movement associated with tremor even at resting states, leading to frequent periods of beta desynchronization. However, their task performance was similar to other patients, suggesting that any impact was minimal.

Another possible limitation is our choice to include 50% unplanned trials as opposed to a lower percentage, such as 30%, which has been used previously and would have made the unplanned stop trials more surprising (Schultz et al., 2023a,b). The use of 50% unplanned stop trials allowed us to limit the total time we required of participants by reducing the total number of trials required without an impact on the number of unplanned stop trials. This choice was also supported by prior work showing similar behavioral effects regardless of percentage of unplanned stop trials (Schultz et al., 2023a). However, it is possible that this adaptation affected the strategies participants employed. That is, given that the probability of unplanned stop trials was identical to planned stop trials, participants may have more consistently engaged proactive inhibitory mechanisms than they otherwise would have.

Finally, our sample had an imbalance in sex between groups (with more females in the HC compared with the PD group) and a relatively small sample size. We addressed this possible confound by controlling for sex in our model to examine behavioral data and demonstrating that the differences between groups were strongest when separating groups by disease state (PD vs HC) and not by sex (male vs female) for the electrophysiology. Nevertheless, a balanced sample in terms of sex would have been optimal.

### Conclusion

One of the major benefits of the CMST is that it allows us to precisely measure stopping behavior and time-lock electrophysiological recording to the moment of stop completion. We were able to leverage this advantage to (1) reveal behavioral deficits in stopping movement in patients on medication, (2) confidently determine that theta occurs early in the stopping process (prior to stopping) and is modulated by task-relevant stimuli (i.e., cue onset) and group (PD ON vs OFF vs HC), and (3) show that beta is modulated by stopping context (i.e., elevated when stopping was unplanned) but that the most prominent beta increase occurs late in the stopping process and in fact, rises after movement termination itself.

## Synthesis

Reviewing Editor: Mikko Airavaara, Helsingin yliopisto - Viikin kampus Decisions are customarily a result of the Reviewing Editor and the peer reviewers coming together and discussing their recommendations until a consensus is reached. When revisions are invited, a fact-based synthesis statement explaining their decision and outlining what is needed to prepare a revision will be listed below. The following reviewer(s) agreed to reveal their identity: NONE. Note: If this manuscript was transferred from JNeurosci and a decision was made to accept the manuscript without peer review, a brief statement to this effect will instead be what is listed below.

In this article, Karekel et al. share their results from a novel experiment using continuous movement termination to explore the cognitive deficits associated with PD and dopaminergic medications. Be analyzing termination of a continuous movement, the authors are able to disentangle the mixed electrophysiological signals associated with most response inhibition tasks. This allows the authors to identify a clear increase in theta power that was associated with informational cues (Start and Stop) as well as a post termination beta rebound. The cue related increases in theta were significantly lower in PD patients (compared to healthy controls) and seem to be suppressed further by dopaminergic medication. This suggests a role for theta oscillations in initiating the motor suppression process and further suggests that alterations in dopaminergic state can compromise this role as evident in the longer stopping times for the PD ON condition. Overall, the manuscript is simple, succinctly presented, and provides novel insight on the mechanisms underlying cognitive control. As such I believe it will be of interest to readers in this field and will make a valuable contribution. I have only minor points below:

In the methods section, the authors mention that one subject misunderstood the instructions and performed most movements counterclockwise. Looking through the methods and figure 1, I was unable to determine how the study participants were told which direction to turn the mouse. This should be clarified in the methods.

The authors provide an interesting interpretation of the post-stopping beta rebound results, suggesting that it may be involved in a "reset" for the subsequent trial. If word count allows, it would also be worth mentioning prior work showing that post response beta rebounds are also increased following error trials. In light of these prior studies, the relatively higher post-response beta rebound following unplanned movements may reflect the recruitment of additional recourses for better cognitive control on the subsequent trial.

## Author Response

Dear Editor, We would like to thank you and Editor and Reviewer for the helpful comments on our manuscript entitled: "Electrophysiological Signatures of Planned and Unplanned Continuous Movement Termination in Parkinson's Disease" We are very glad that the reviewer found our manuscript to be "simple, succinctly presented", and that it provides "novel insight on the mechanisms underlying cognitive control". Below we have addressed each point made by the reviewer. These changes to the manuscript have been indicated with yellow highlighting. We also corrected a few typographical errors and added a few citations. We believe that with these changes our manuscript is much improved.

We thank you for your renewed consideration.

Sincerely, Nicole C. Swann, PhD and Apoorva Karekal MS University of Oregon Department of Human Physiology DEPARTMENT OF HUMAN PHYSIOLOGY 1240 University of Oregon, Eugene OR 97403-1240 T (541) 346-4107 F (541) 346-2841 An equal-opportunity, affirmative-action institution committed to cultural diversity and compliance with the Americans with Disabilities Act Reviewer #1:

1. In the methods section, the authors mention that one subject misunderstood the instructions and performed most movements counterclockwise. Looking through the methods and figure 1, I was unable to determine how the study participants were told which direction to turn the mouse. This should be clarified in the methods.

We agree this was an omission. We have now clarified that the intended direction of mouse movements was explained during the task explanation in the Methods section and in the Figure 1 legend describing the task paradigm. Additionally, participants were provided trial by trial feedback during the training period (including if they were moving the mouse in the incorrect direction). While one participant was inconsistent in their mouse-movement direction, they otherwise did the task correctly, so their data were retained.

The relevant section added is reproduced below: " During this training participants were instructed to move in a clockwise direction and got feedback after every trial if they moved in the wrong direction (i.e, anticlockwise). " "Participants were instructed to start moving a cursor controlled by a computer mouse in the clockwise direction" 2. The authors provide an interesting interpretation of the post-stopping beta rebound results, suggesting that it may be involved in a "reset" for the subsequent trial. If word count allows, it would also be worth mentioning prior work showing that post response beta rebounds are also increased following error trials. In light of these prior studies, the relatively higher post-response beta rebound following unplanned movements may reflect the recruitment of additional recourses for better cognitive control on the subsequent trial.

The Reviewer brings up an excellent point. This is indeed another (non-mutually exclusive) interpretation of our findings. We have incorporated this possible interpretation into the Discussion section.

The relevant section added is reproduced below: "Another interpretation (not necessarily mutually exclusive) is that the late increase in beta may reflect increased engagement of cognitive control following unplanned stop trials which may manifest as increased caution for the subsequent trial. This may be akin to the modulations in beta which have been observed following errors https://www.zotero.org/google-docs/?x06rdJ . For future analysis, it would be interesting to explore this phenomenon where the trial types can be divided based on prior trial, that is, post response beta power for trial preceded by an unplanned versus planned stop. " Again, we thank the Reviewer for their helpful comments which have improved our manuscript.
