# Supplementary material for: Electrophysiological Signatures of Planned and Unplanned Continuous Movement Termination in Parkinson’s Disease
Source: eNeuro. 2025 Oct 28;12(10):ENEURO.0286-25.2025. doi: 10.1523/ENEURO.0286-25.2025 (PMC12570292; doi:10.1523/ENEURO.0286-25.2025)
Supplement: Figure 2-1 — Table showing p-values for comparison between groups and conditions for SCT. Asterisk denotes significance (p < 0.05, FDR corrected). Download Figure 2-1, DOCX file. [file eneuro-12-ENEURO.0286-25.2025-s001.docx]

| **Groups** | **Condition comparison** | **P value** |
| --- | --- | --- |
| HC S1 | Plan vs Unplan | *7.110×10^−9^ |
| HC S2 | Plan vs Unplan | *2.788×10^−8^ |
| PD OFF | Plan vs Unplan | *7.719×10^−8^ |
| PD ON | Plan vs Unplan | *8.702×10^−8^ |
|  |  |  |
| **Group comparisons** | **Condition** | **P value** |
| HC S1 - PD OFF | Plan | 0.241 |
| HC S1 - PD OFF | Unplan | 0.286 |
| HC S2 - PD ON | Plan | *0.049 |
| HC S2 - PD ON | Unplan | *0.049 |
| PD OFF - PD ON | Plan | *0.0039 |
| PD OFF - PD ON | Unplan | *0.0044 |

**Extended Data Figure 2-1:** Table showing p-values for comparison between groups and conditions for SCT. Asterisk denotes significance (p<0.05, FDR corrected).
