# Supplementary figures and images for: Electrophysiological Signatures of Planned and Unplanned Continuous Movement Termination in Parkinson’s Disease
Source: eNeuro. 2025 Oct 28;12(10):ENEURO.0286-25.2025. doi: 10.1523/ENEURO.0286-25.2025 (PMC12570292; doi:10.1523/ENEURO.0286-25.2025)

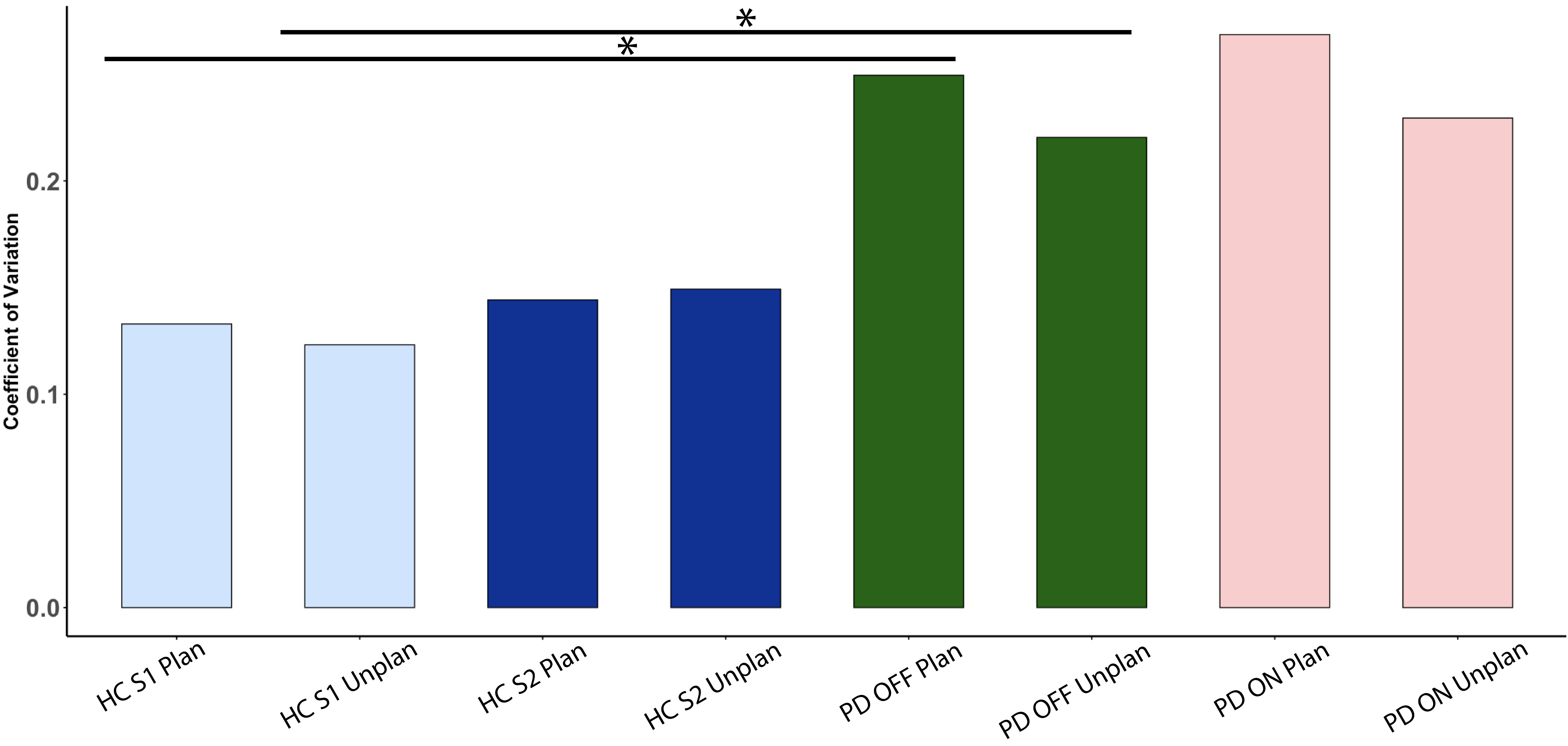

Supplement: Figure 2-2 — Coefficient of variation (CV) across groups and conditions for SCT. Black lines with an asterisk denote significance (p < 0.05, FDR corrected). The SCT was more variable for PD OFF than HC S1 groups for SCT for both planned and unplanned stopping. Download Figure 2-2, TIF file. [file eneuro-12-ENEURO.0286-25.2025-s002.tif]

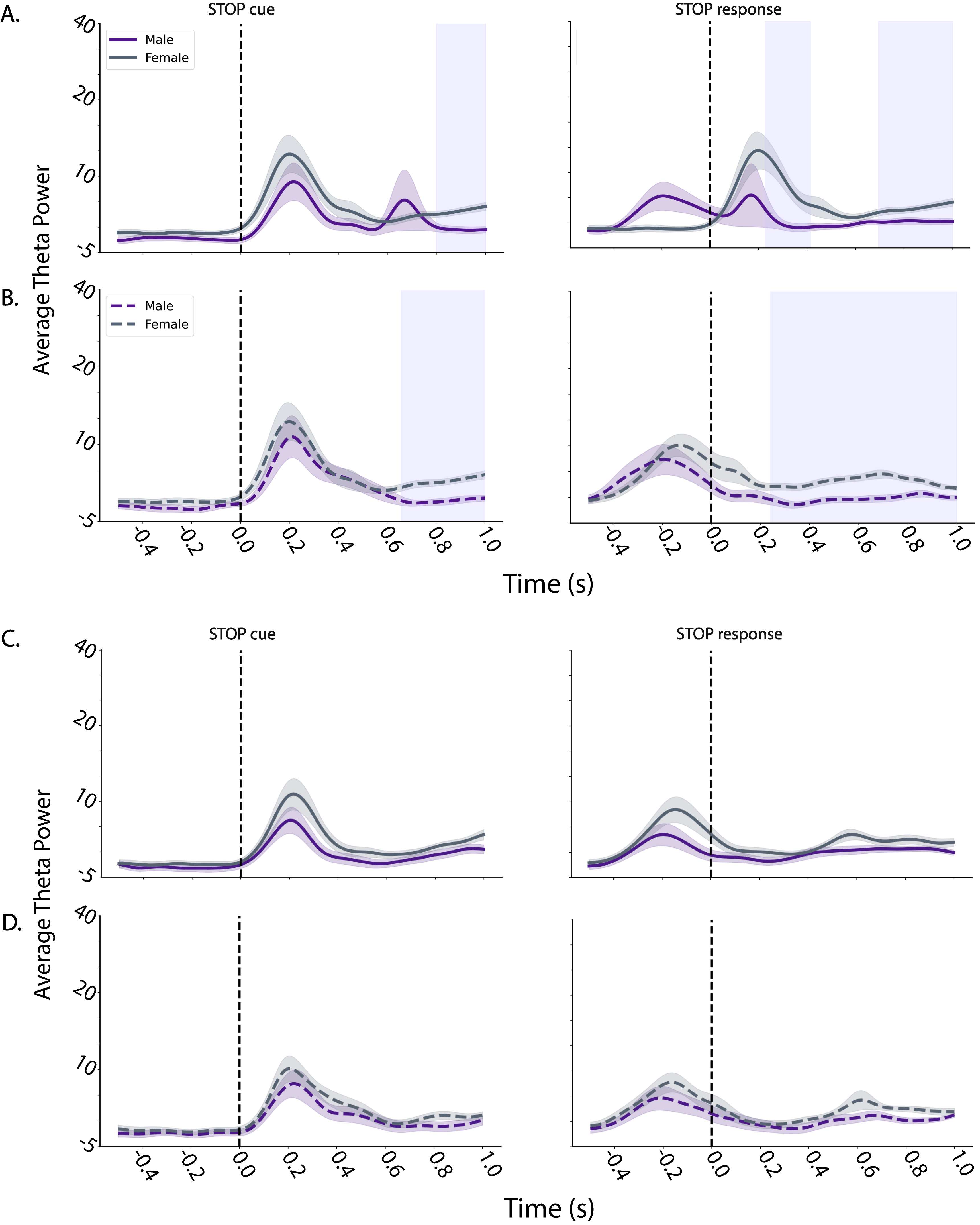

Supplement: Figure 3-1 — Average theta power in midfrontal cortex comparing between males and females across groups. Time-locked to both the stop cue (“STOP Cue”, left) and time of actual motor cessation (“STOP response”, right). Theta traces comparing male and females across HC S1 and PD OFF groups for planned (A) and unplanned (B) conditions. Theta trace comparing male and females across HC S2 and PD ON groups for planned (C) and unplanned (D) conditions. For each analysis, time zero is indicated with a dashed line. Light blue shaded regions show time periods that are significant (p < 0.05, cluster-based time correction for multiple comparison). The shaded area region surrounding each power trace represents standard error. Theta differences between males and females exist, but compared to the HC S1 and PD OFF groups the differences persist over shorter time windows and mostly occur well after the cue or response. Download Figure 3-1, TIF file. [file eneuro-12-ENEURO.0286-25.2025-s004.tif]

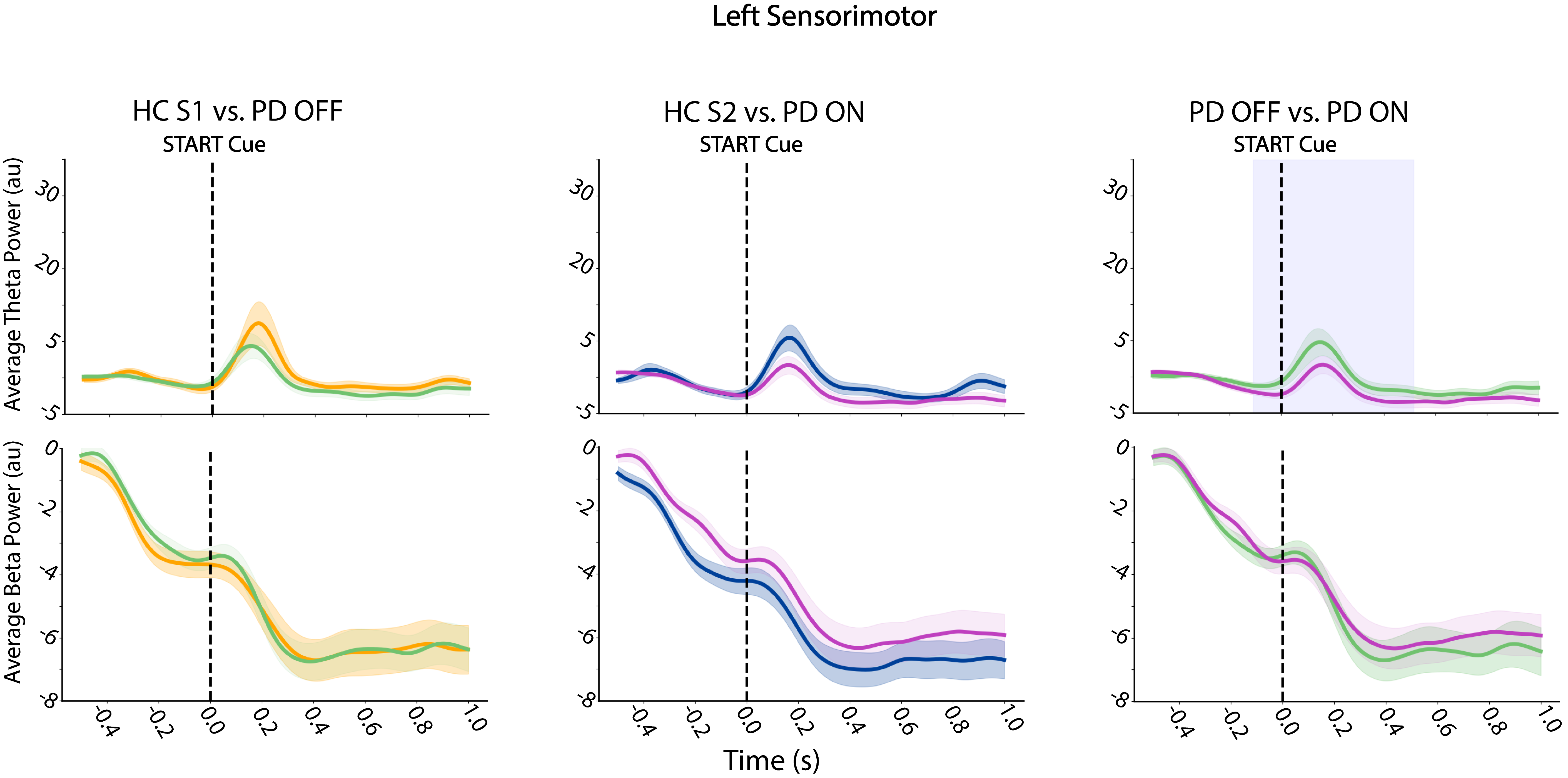

Supplement: Figure 7-1 — Average theta and beta power for movement initiation in left sensorimotor cortex. Average theta power (top row) and beta power (bottom row) compared across groups aligned to start cue. For each analysis, time zero is indicated with a dashed line. Blue shaded regions show time periods that are significant (p < 0.05, cluster- based time correction for multiple comparison). The shaded area region surrounding each power trace represents standard error. Overall theta power in the left sensorimotor region is similar to the midfrontal region, but of lower amplitude than the midfrontal cortex for all groups. Sensorimotor beta power decreases following the signal to move, as expected (Pfurtscheller, 1981), but does not differ between groups. Download Figure 7-1, TIF file. [file eneuro-12-ENEURO.0286-25.2025-s006.tif]
