# Supplementary material for: Electrophysiological Signatures of Planned and Unplanned Continuous Movement Termination in Parkinson’s Disease
Source: eNeuro. 2025 Oct 28;12(10):ENEURO.0286-25.2025. doi: 10.1523/ENEURO.0286-25.2025 (PMC12570292; doi:10.1523/ENEURO.0286-25.2025)
Supplement: Figure 2-3 — Table showing p-values for group and condition comparisons of CV for SCT. Asterisk denotes significance (p < 0.05, FDR corrected). Download Figure 2-3, DOCX file. [file eneuro-12-ENEURO.0286-25.2025-s003.docx]

| **Group comparisons** | **Condition** | **P value** |
| --- | --- | --- |
| HC S1 - PD OFF | Plan | *0.023 |
| HC S1 - PD OFF | Unplan | *0.024 |
| HC S2 - PD ON | Plan | 0.052 |
| HC S2 - PD ON | Unplan | 0.079 |
| PD OFF - PD ON | Plan | 0.478 |
| PD OFF - PD ON | Unplan | 0.525 |

**Extended Data Figure 2-3:** Table showing p-values for group and condition comparisons of CV for SCT. Asterisk denotes significance (p<0.05, FDR corrected).
