# Supplementary material for: Electrophysiological Signatures of Planned and Unplanned Continuous Movement Termination in Parkinson’s Disease
Source: eNeuro. 2025 Oct 28;12(10):ENEURO.0286-25.2025. doi: 10.1523/ENEURO.0286-25.2025 (PMC12570292; doi:10.1523/ENEURO.0286-25.2025)
Supplement: Figure 6-1 — Table showing p-values for comparison of theta and beta peak for each group and condition. Asterisk denotes significance (p < 0.05, FDR corrected). Download Figure 6-1, DOCX file. [file eneuro-12-ENEURO.0286-25.2025-s005.docx]

| **Group comparisons** | **Condition** | **P value** |
| --- | --- | --- |
| HC S1 | Plan | *7.591 × 10^-11^ |
|  | Unplan | *1.881 × 10^-11^ |
| HC S2 | Plan | *7.591 × 10^-11^ |
|  | Unplan | *2.972 × 10^-10^ |
| PD OFF | Plan | *2.978 × 10^-6^ |
|  | Unplan | *3.349 × 10^-10^ |
| PD ON | Plan | *7.091 × 10^-7^ |
|  | Unplan | *8.565 × 10^-9^ |

**Extended Data Figure 6-1:** Table showing p-values for comparison of theta and beta peak for each group and condition. Asterisk denotes significance (p<0.05, FDR corrected).
